# The occurrence of hyponatremia and its importance as a prognostic factor in a cross-section of cancer patients

**DOI:** 10.1186/s12885-016-2610-9

**Published:** 2016-07-29

**Authors:** Jorge J. Castillo, Ilya G. Glezerman, Susan H. Boklage, Joseph Chiodo, Beni A. Tidwell, Lois E. Lamerato, Kathy L. Schulman

**Affiliations:** 1 Dana-Farber Cancer Institute, 450 Brookline Ave, M221, Boston, MA 02215 USA; 2 Memorial Sloan-Kettering Cancer Center, New York, NY USA; 3 Otsuka America Pharmaceutical, Inc, Princeton, NJ USA; 4 Outcomes Research Solutions, Inc, Waltham, MA USA; 5 Henry Ford Health System, Detroit, USA

**Keywords:** Hyponatremia, Euvolemic, Hypervolemic, Cancer, Survival

## Abstract

**Background:**

Hyponatremia is prognostic of higher mortality in some cancers but has not been well studied in others. We used a longitudinal design to determine the incidence and prognostic importance of euvolemic and hypervolemic hyponatremia in patients following diagnosis with lymphoma, breast (BC), colorectal (CRC), small cell lung (SCLC), or non-small cell lung cancer (NSCLC).

**Methods:**

Medical record and tumor registry data from two large integrated delivery networks were combined for patients diagnosed with lymphoma, BC, CRC, or lung cancers (2002–2010) who had ≥1 administration of radiation/chemotherapy within 6 months of diagnosis and no evidence of hypovolemic hyponatremia. Hyponatremia incidence was measured per 1000 person-years (PY). Cox proportional hazard models assessed the prognostic value of hyponatremia as a time-varying covariate on overall survival (OS) and progression-free survival (PFS).

**Results:**

Hyponatremia incidence (%, rate) was 76 % each, 1193 and 2311 per 1000 PY, among NSCLC and SCLC patients, respectively; 37 %, 169 in BC; 64 %, 637 in CRC, and 60 %, 395 in lymphoma. Hyponatremia was negatively associated with OS in BC (HR 3.7; *P* = <.01), CRC (HR 2.4; *P* < .01), lung cancer (HR 2.4; *P* < .01), and lymphoma (HR 4.5; *P* < .01). Hyponatremia was marginally associated with shorter PFS (HR 1.3, *P* = .07) across cancer types.

**Conclusions:**

The incidence of hyponatremia is higher than previously reported in lung cancer, is high in lymphoma, BC, and CRC and is a negative prognostic indicator for survival. Hyponatremia incidence in malignancy may be underestimated. The effects of hyponatremia correction on survival in cancer patients require further study.

## Background

Hyponatremia, the most common electrolyte disturbance in hospitalized patients, results from loss of body sodium or potassium with secondary water retention (hypovolemic); from relative or absolute excess of body water (euvolemic, including syndrome of inappropriate antidiuretic hormone secretion (SIADH)); and from edema formation due to renal sodium and water retention (hypervolemic) [1, 2]. Hypovolemic hyponatremia responds readily to volume repletion, while treatment modalities in euvolemic and hypervolemic hyponatremia are not well standardized [1]. Hyponatremia incidence and prevalence vary greatly depending on the population, the presence and type of malignancy, clinical setting, and serum sodium cutoff point [3–5]. Its prevalence has been reported in 1.7 % of the general United States (US) population and in 3.4 % of respondents who identified themselves as having cancer [2]. Hyponatremia incidence in cancer patients has been reported in as many as 47 % of hospital admissions, [6] and the frequency of moderate to severe hyponatremia in hospitalized patients can range from 24 to 50 %, depending on malignancy type [7].

To date, most studies of hyponatremia in cancer have been performed primarily in hospitalized patients or in patients after the occurrence of another clinical event, eg, surgical resection, chemotherapy initiation [6–9]. These studies have largely been conducted in patients with lung or hematologic cancers or as an analysis of multiple cancer types in studies assessing the prognostic effects of hyponatremia. However, little research has been conducted in other highly prevalent cancers, such as breast or colorectal cancer. Moreover, to our knowledge, no study has examined the frequency and prognostic impact of hyponatremia longitudinally, beginning with the date of cancer diagnosis. The current study assessed the incidence and prognostic importance of euvolemic and hypervolemic hyponatremia on or after diagnosis with breast cancer (BC), colorectal cancer (CRC), small cell lung cancer (SCLC), non-SCLC (NSCLC), and lymphoma (Hodgkins, non-Hodgkins).

## Methods

### Study design

This retrospective cohort analysis combined medical record and tumor registry data from two large, integrated delivery networks (IDN) serving patients in the Midwest (IDN 1) and MidAtlantic (IDN 2) regions of the US. Both are not-for-profit, physician-led IDNs, which together contain data for more than 7 million patients. Patient anonymity and confidentiality were preserved by de-identification of the database in compliance with the Health Insurance Portability and Accountability Act of 1996. For IDN 1, the protocol was approved by an institutional review board (IRB) and for IDN 2, the production and delivery of de-identified data was deemed exempt from IRB review.

### Patients

Patients selected into the study were adults with BC, CRC, SCLC, NSCLC, or lymphoma documented in their respective cancer registry between December 1, 2002 and November 30, 2010 (IDN 1) or January 1, 2005 and December 31, 2009 (IDN 2), provided that the cancer stage was known, the patient met analytic case requirements, and had ≥1 administration of radiation or chemotherapy ≤6 months of diagnosis. In addition, patients were required to meet continuous enrollment thresholds in IDN1 (12 months prior to and ≥1 month post cancer diagnosis) or continuous clinical activity thresholds in IDN 2 (≥1 in-system contacts in the 12 months prior to and ≥3 in the 6 months post cancer diagnosis). Patients who had insufficient or conflicting documentation in their medical records, had registration of a non-invasive tumor, received cancer-related therapy outside of the IDN, or had hypovolemic hyponatremia were excluded. Patients were followed until study end, death, clinical trial entry, new primary cancer onset, disenrollment (IDN 1), or end of continuous clinical activity (IDN 2).

### Analysis

The cohort was divided into patients who developed one or more episodes of hyponatremia at any time during follow-up and those who never developed hyponatremia during follow-up. Hyponatremia, defined as a serum sodium laboratory result ≤135 mEq/L, was captured as a time-varying covariate since it could resolve and then reoccur. A hyponatremia episode began on the first abnormal test result date and was considered resolved on the first of 2 subsequent normal results. Hyponatremia incidence was measured per 1000 person years (PY) of observation and reported with 95 % confidence intervals (CIs).

Hyponatremia was classified as mild (131–135 mEq/L), moderate (125–130 mEq/L), or severe (<125 mEq/L) based on the lowest observed serum sodium value during the episode and was then further classified as euvolemic, hypervolemic, or hypovolemic based on a multi-stage algorithm using existing electronic laboratory data, medication orders, and ICD-9-CM diagnosis files. The first stage of the algorithm, which has not yet been validated, identified cases of true hyponatremia based on serum osmolality test results of <275 mOsm/kg ≤48 h of the serum sodium result with no evidence of hyperglycemia. The algorithm then divided patients into hypovolemic, hypervolemic or euvolemic hyponatremia decision trees based on ICD 9 CM diagnosis codes, disease history, and urine osmolality values. The algorithm further segmented euvolemia into “SIADH,” largely determined by laboratory values, and “other euvolemic hyponatremia,” assigned to patients that did not meet the criteria for hypervolemic but had a history of hypothyroidism, adrenal insufficiencies, psychogenic polydipsia, or diuretic use.

Patient demographics were captured as of the date of cancer diagnosis. Baseline clinical characteristics were captured during the 12 months prior to cancer diagnosis. A 3-point universal performance status score (PS) combined Eastern Cooperative Oncology Group (ECOG) and Karnofsky Performance Status (KPS) scores [10]. Grade 1 PS (good) was comprised of ECOG PS 0–1 and KPS 80–100; Grade 2 PS (fair) of ECOG PS 2 and KPS 60–70; and Grade 3 PS (poor) of ECOG PS 3–4 and KPS 10–60. The statistical significance of between-cohort differences in categorical variables was evaluated using the chi-square test. Continuous data were compared using the *t*-test. All tests were two-tailed, with a significance level of *p* < 0.05.

The primary study outcome was overall survival (OS). Mortality was ascertained from registry records and state death records. The secondary study outcome, progression free survival (PFS), was recorded and reported for IDN1 only due to resource constraints. The definition for solid tumor progression, modified from RECIST v1.1., [11] included: recurrence in a disease-free person, stage progression in a patient with active disease, increase in existing lesion size, occurrence of a new lesion, and “other.” Disease progression in lymphoma, using Cheson criteria, [12] included: occurrence of a new lesion, increase in positron emission tomography uptake, increase in lymph node or lesion size, recurrence in a disease free person, and “other.”

Survival in days was calculated separately for OS and PFS, from the date of cancer diagnosis to the date of all cause death (OS) or progression (PFS) in patients with the event and until the first evidence of censoring or study end for patients who were not known to have died or to have experienced progression by the end of the study. Kaplan-Meier life tables were used to estimate survival at 1, 3, and 5 years. A Cox Proportional Hazard model with hyponatremia as a time-varying covariate was employed to identify the independent prognostic factors associated with an increased risk of death across all cancer types and among patients in each individual cancer type.

## Results

### Patients

**Fig. 1:**
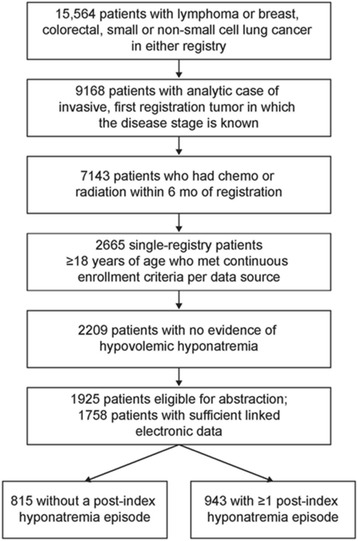
Study flow chart

During accrual of the study sample (detailed in Fig. 1), 1758 patients met all study requirements from a pool of 15,564 patients in both IDNs. It should be noted that 456 patients with hypovolemic hyponatremia (3 %) were excluded from the study because this type of hyponatremia generally responds to treatment with intravenous fluids, while hypervolemic and euvolemic hyponatremia tend to be more difficult to diagnose and treat [1, 4, 13]. Additionally, intravenous hydration is often required for many cancer therapies and its use may complicate analysis in patients with hypovolemic hyponatremia [4]. Among study-eligible patients, 71 % were female, with a mean (SD) age of 60 (13.0) years and a mean (SD) follow-up duration of 3.1 (2.7) years. Selected characteristics of the study population are shown in Table 1. Patients who developed hyponatremia on or after cancer diagnosis were more likely to be male, white, and have a shorter follow-up time (Table 1). They were also significantly more likely to have lung cancer or CRC and less likely to have BC. Across tumor types, the hyponatremic cohort was more likely to have metastatic disease and a worse performance status after cancer diagnosis.

**Table 1:** Demographic and clinical characteristics

Number of patients	Total	No hyponatremia episode	≥1 hyponatremia episode	*P* value
	*N* = 1758	*n* = 815	*n* = 943	
Demographic characteristics				
Sex, n (%)				<0.01
Male	503 (29)	145 (18)	358 (38)	
Female	1255 (71)	670 (82)	585 (62)	
Age group collapsed, n (%)				0.06
18–64	1108 (63)	533 (65)	575 (61)	
≥65	650 (37)	282 (35)	368 (39)	
Mean age (SD)	60.2 (13)	59.6 (13)	60.6 (13)	0.11
Race, n (%)				0.09
Asian	18 (1)	8 (1)	10 (1)	
Black	340 (19)	179 (22)	161 (17)	
White	1393 (79)	627 (77)	766 (81)	
Median household income, n (%)				0.16
≤$49,999	1030 (59)	480 (59)	550 (58)	
$50,000–$69,999	460 (26)	197 (24)	263 (28)	
≥$70,000	239 (14)	123 (15)	116 (12)	
Diagnosis year, n (%)				0.02
2002–2004	270 (15)	146 (18)	124 (13)	
2005–2007	869 (49)	386 (47)	483 (51)	
2008–2010	619 (35)	283 (35)	336 (36)	
Mean length of follow-up, y (SD)	3.1 (3)	3.3 (3)	3.0 (3)	0.03
Clinical characteristics at baseline				
Cancer type, n (%)				
Breast	839 (48)	533 (65)	306 (32)	<0.01
Colorectal	233 (13)	84 (10)	149 (16)	<0.01
Colon	146 (8)	50 (61)	96 (10)	<0.01
Rectal	87 (5)	34 (4)	53 (6)	0.16
Lung	485 (28)	117 (14)	368 (39)	<0.01
Small cell	80 (5)	19 (2)	61 (7)	<0.01
Non-small cell	405 (23)	98 (12)	307 (33)	<0.01
Lymphoma	201 (11)	81 (10)	120 (13)	0.07
Hodgkins	29 (2)	12 (2)	17 (2)	0.59
Non-Hodgkins	172 (10)	69 (9)	103 (11)	0.08
Distant metastasis, n (%)	384 (22)	99 (12)	285 (30)	<0.01
Any PS within 90 days of diagnosis, n (%)	864 (49)	384 (47)	480 (51)	0.11
Grade 1: ECOG 0, 1; KPS 80–100^a^	747 (87)	350 (91)	397 (83)	<0.01
Grade 2: ECOG 2; KPS 60–70^a^	78 (9)	26 (7)	52 (11)	0.04
Grade 3: ECOG 3, 4; KPS 10–50^a^	39 (5)	8 (2)	31 (7)	<0.01
Clinical characteristics during follow-up				
Distant metastasis, n (%)	513 (29)	120 (15)	393 (42)	<0.01
PS, last observed documentation, n (%)	1249 (71)	553 (68)	696 (74)	<0.01
Grade 1: ECOG 0, 1; KPS 80–100^a^	990 (79)	499 (90)	491 (71)	<0.01
Grade 2: ECOG 2; KPS 60–70^a^	141 (11)	34 (6)	107 (15)	<0.01
Grade 3: ECOG 3, 4; KPS 10–50^a^	118 (9)	20 (4)	98 (14)	<0.01
Hospice services, n (%)	129 (7)	21 (3)	108 (12)	<0.01
First course surgical resection, n (%)	1029 (62)	563 (72)	466 (52)	<0.01
Any chemo and hormonal therapies, n (%)	1410 (80)	595 (73)	815 (86)	<0.01
Alkylating agents^b^	547 (39)	269 (45)	278 (34)	<0.01
Antimetabolites^b^	427 (30)	113 (19)	314 (39)	<0.01
Antitumor antibiotics^b^	452 (32)	223 (38)	229 (28)	<0.01
Hormone therapy^b^	594 (42)	318 (53)	276 (34)	<0.01
Mitotic inhibitors^b^	761 (54)	260 (44)	501 (62)	<0.01
Platinum agents^b^	512 (36)	114 (19)	398 (49)	<0.01
Targeted therapies^b^	375 (27)	124 (21)	251 (31)	<0.01
Other^c^	175 (10)	48 (6)	127 (13)	<0.01

*Abbreviations*: *ECOG* Eastern Cooperative Oncology Group, *KPS* Karnofsky PS, *PS* performance status, *SD* standard deviation, *y* year

^a^Percent of patients with any PS

^b^Percent of patients with any chemo or hormonal therapy

^c^Other treatments including immunotherapies and topoisomerases

### Hyponatremia incidence

**Fig. 2:**
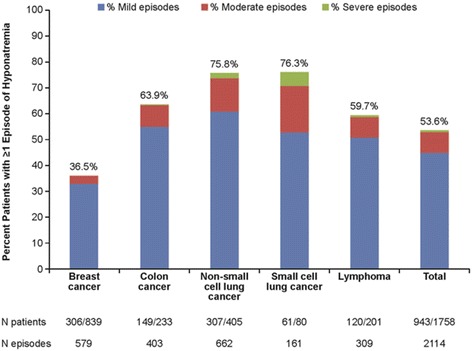
Proportion of patients with hyponatremia, by hyponatremia severity and cancer type

Across cancer types, 54 % had ≥1 episode of euvolemic or hypervolemic hyponatremia episode (Fig. 2). The frequency of hyponatremia was highest among patients with NSCLC and SCLC (76 % each), and lowest among patients with BC (37 %). The majority (84 %) of all hyponatremia episodes were mild. The incidence rate (IR) of hyponatremia per 1000 PY was 385.5 (95 % CI, 369.2–402.2), with individual rates of 169 (BC), 395 (lymphoma), 637 (CRC), 1193 (NSCLC), and 2311 (SCLC). The mean (SD) number of hyponatremia episodes per patient was 2.2 (1.9), ranging from a low of 1.9 in BC to a high of 2.7 in CRC. Median time to first hyponatremia episode was 59 days, ranging from a low of 10 days in SCLC to a high of 194 days in BC. Median duration of each hyponatremia episode was 16 days.

Across all cancer types, 284 patients (16 %) had ≥1 moderate or severe episode of hyponatremia. Moderate or severe episodes occurred in 6 % of BC patients, 19 % of both CRC and lymphoma patients, 27 % of NSCLC patients, and 46 % of SCLC patients. Among patients with ≥1 moderate or severe hyponatremia episode, 58 % of hyponatremia episodes were mild, 37 % were moderate, and 6 % were severe. The mean (SD) number of hyponatremia episodes per patient was 2.9 (2.4), ranging from 2.4 for both BC and NSCLC to 3.9 for CRC. Median time to first hyponatremia episode was 19 days, ranging from 4 days for SCLC to 105 days for BC. Mean duration of each hyponatremia episode ranged from 41 days for patients with SCLC to 130 days for patients with BC.

### Survival analysis

**Fig. 3:**
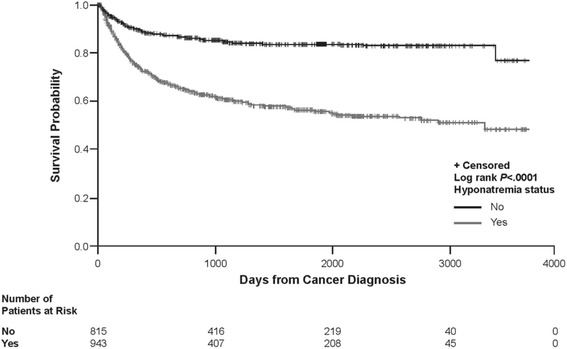
Kaplan Meier plot of overall survival across cancer types

**Fig. 4:**
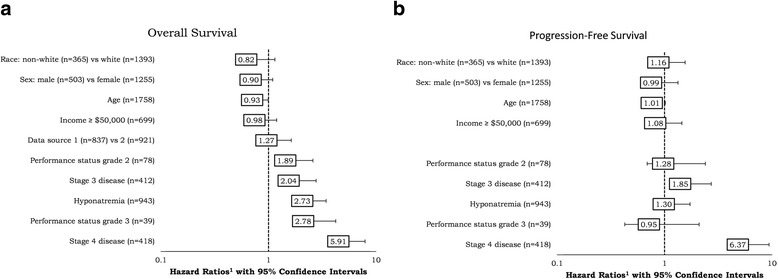
Overall survival and progression-free survival across cancer types

Across the studied cancer types, 27 % of patients died during follow-up. SCLC patients had the highest proportion of deaths at 86 % whereas BC patients had the lowest at 5 %. Life table data presented in Table 2 characterizes OS, by cancer type, at 1, 3, and 5 years. The Kaplan-Meier overall survival curves, across all cancer types, are shown in Fig. 3. Cox model results are presented graphically in Fig. 4a and 4b. Experiencing one or more episodes of hyponatremia was associated with a significant increase in the likelihood of death (HR 2.7, 95 % CI, 2.2–3.4; *P* < 0.01), as was having stage 3 (HR 2.0, 95 % CI, 1.5–2.7; *P* < 0.01) or stage 4 disease at diagnosis (HR 5.9, 95 % CI, 4.4–7.9; *P* < 0.01), having a fair/poor PS score at diagnosis (HR 2.8, 95 % CI, 1.8–4.2; *P* < 0.01), or having an unknown PS at the time of diagnosis (HR 1.5, 95 % CI, 1.2–1.8; *P* < 0.01). Developing hyponatremia was associated with significantly increased likelihood of death in each cancer specific model, except for SCLC: BC (HR 3.7, 95 % CI, 1.9–7.2; *P* < 0.01), CRC (HR 2.4, 95 % CI, 1.3–4.7; *P* < 0.01), lung cancer (HR 2.4, 95 % CI, 1.8–3.2; *P* < 0.01), SCLC (HR 1.5, 95 % CI 0.82–2.8; *P* = 0.19), NSCLC (HR 2.8, 95 % CI 2.0–3.9; *P* < 0.01) and lymphoma (HR 4.5, 95 % CI, 1.8–11.5; *P* < 0.01) (Fig. 4).

**Table 2:** Life tables depicting overall survival at 1, 3, and 5 years

Cancer type	1 year			3 year			5 year		
OS, %	All	HN	No HN	All	HN	No HN	All	HN	No HN
All cancer types	81	64	87	72	49	81	69	45	79
Breast cancer	98	95	99	97	92	97	94	89	95
Colorectal cancer	86	79	90	73	65	78	68	56	76
Colon cancer	85	78	88	69	62	72	63	53^a^	72
Rectal cancer	87	80	92	81	68	89	75	62^a^	85^a^
Lung cancer	45	39	51	22	16	29	17	11^a^	24
Non-small cell	49	39	56	26	18	32	19	12^a^	27
Small cell	30	40	18^a^	6^a^	6^a^	8^a^	6^a^	6^a^	8^a^
Lymphoma	87	79	92	84	71	91	82	67	91
Hodgkins	100	100^a^	100	96	88^a^	100	96^a^	88^a^	100^a^
Non-Hodgkins	85	76	91	82	68	89	80	64	89

*Abbreviation*: *HN* hyponatremia

^a^Effective sample size for the year in question is ≤10 patients

Twenty-five percent (*n* = 228) of patients in IDN 1 experienced disease progression during follow-up, ranging from a low of 10 % in BC to a high of 65 % in SCLC. Mean (SD) time to progression was 395 (512) days, shortest in SCLC patients at 160 days and longest in patients with BC at 763 days. PFS at 1, 3, and 5 years was 87, 81, and 78 %, respectively. Cox model results are presented in Fig. 3. Experiencing one or more episodes of hyponatremia was not associated with a significant change in PFS (HR 1.3, 95 % CI, 0.98–1.7; *P* = 0.07); however, patients with stage 3 (HR 1.8 95 % CI, 1.3–2.7; *P* < 0.01), or stage 4 cancer at diagnosis (HR 6.4 95 % CI, 4.3–9.4; *P* < 0.01) were at increased likelihood to experience disease progression.

## Discussion

This study combined administrative and medical record data from two large healthcare delivery systems in the US to ascertain the incidence of hypervolemic or euvolemic hyponatremia after cancer diagnosis and to assess its prognostic importance on OS and PFS. Study findings suggest that the incidence of hyponatremia among patients with NSCLC and SCLC is higher than previously reported, that the incidence of hyponatremia in BC, CRC, and lymphoma is high, and that the occurrence of hyponatremia in all 4 types of cancer is a negative prognostic indicator.

The incidence of hyponatremia in cancer patients varies greatly depending on cancer type, clinical setting, and the serum sodium threshold employed [3–5, 14]. Malignancy-related SIADH due to ectopic secretion of arginine vasopressin manifesting as euvolemic hyponatremia is most commonly seen in patients with SCLC, but can also be associated with other malignancy types [3–5]. In addition, antineoplastic and cancer therapy palliative drugs are also known to cause hyponatremia and many are directly associated with SIADH [3–5]. Other underlying conditions, such as pain and nausea, or routine hospital treatments may also cause hyponatremia, contributing to disease complexity.

Study findings suggest that the hyponatremia incidence among patients with lung cancer is higher than previous reported. Hyponatremia occurred in 76 % of lung cancer patients in the current study, considerably higher than 20–50 %, as previously reported [7, 15–18]. This difference in incidence may be greater than observed because the current study excluded patients with hypovolemic hyponatremia, while previously published studies did not. However, previous studies also characterized hyponatremia incidence upon the occurrence of a specific clinical event such as hospitalization, surgical resection or chemotherapy. As such, the measurement of hyponatremia in these studies did not include hyponatremia in patients who did not experience the study-qualifying event (eg resection), or who experienced hyponatremia prior to the qualifying event. Differences in incidence between SCLC and NSCLC subgroups did exist in the current study. Forty-six percent of SCLC patients experienced an episode of moderate/severe hyponatremia (vs 27.4 % NSCLC) with the IR per thousand PY almost twice as high among SCLC patients (2311 vs 1193).

Results from the current study also suggest that hyponatremia incidence in patients with CRC, lymphoma, and BC is noteworthy, occurring in 64, 60, and 36 % of patients at an IR per 1000 PY of 637, 395, and 169, respectively. While most hyponatremia episodes in these patients were mild, moderate to severe hyponatremia occurred in 19 % of CRC and lymphoma patients and in 6 % of BC cases. As was observed in lung cancer, hyponatremia incidence is higher in this study than has been previously reported, ie, 24 % of BC, 27 % of lymphoma and 28 % of CRC patients [7, 19].

Hyponatremia has been correlated with shorter survival in a number of studies, although too few studies have been conducted in a given cancer type to support meta-analyses [3, 7, 17–21]. The current study adds to the growing body of literature in lung cancer and lymphoma, and helps to establish preliminary results in CRC and BC. Current study findings confirm the prognostic importance of hyponatremia in lung cancer. The hazard ratio (95 % CI, *P* value) associated with hyponatremia in the OS lung cancer model was 2.4 (1.8–3.2, *P* < 0.01). Findings in the SCLC specific model did not reach statistical significance, but these were constrained by sample size. Findings in the NSCLC-specific model were significant and are generally higher than those previously reported [3, 21]. The current study is also one of the first to establish the prognostic importance of hyponatremia on OS in lymphoma, CRC, and BC. A recent CRC study concluded that patients with mild (HR 1.7), moderate (HR 2.2), and severe (HR 2.2) hyponatremia upon hospitalization had significantly shorter survival (*P* < 0.001) [19]. These findings are also consistent with a recent meta-analysis which evaluated the prognostic importance of the correction of hyponatremia across a variety of clinical conditions, including all forms of malignancy [22].

Study findings also suggest that hyponatremia may impact PFS. However, PFS was collected only at a single research site and model development, across cancer types, was constrained by sample size and number of events. However, our results are consistent with a study by Tiseo et al. of hyponatremia in SCLC, in which PFS in the univariate model did not meet significance, but did show a trend of correlation between hyponatremia and PFS (HR = 1.23, 95 % CI 0.97–1.55; *P* = 0.085) [21].

Although hyponatremia is associated with a poorer prognosis in cancer patients, as in other diseases, there are still questions as to whether hyponatremia is a marker of disease severity, as evidenced in studies in palliative-care patients, [8, 23] or if correction of hyponatremia can lead to overall patient benefits, including survival [24–26]. A recent meta-analysis has suggested that correction of hyponatremia improves survival, particularly in patients who are corrected >130 mEq/L [22]. Additionally, findings from a subsequent study suggest that correction of sodium level in cancer patients with severe hyponatremia facilitates additional treatment, and results in significantly greater OS, although the authors note that a causal relationship could not be established [20]. Little is known about the actual mechanism by which hyponatremia influences a poorer prognosis. Underlying renal and/or endocrine dysfunction, more aggressive biological behavior of cancer cells that produce antidiuretic hormone (ADH), and the effects of higher than normal levels of ADH overall are all plausible potential explanations. Although our study suggests that hyponatremia is an adverse prognostic factor in a multivariate statistical analysis, it is unclear if hyponatremia is the result of multiple pathophysiological effects, or an independent biological factor. Additional research is needed to further elucidate these theories.

While the study sample was comparatively large, it was not a random sample and the sources of the data are worth reviewing. Although IDN1 and IDN2 each represent geographically constrained areas, they represent care delivered by some of the largest and best delivery networks within the US. Results, as such, may not generalize to care provided in other areas of the US, from smaller delivery networks or those not associated with academic medical centers. The IDN1 sample only included members of their wholly owned insurance plan and excluded Medicaid patients and the uninsured. While IDN2 patients were not restricted based on payer, it is possible that data capture may have been incomplete if out of network care was not documented. In addition, the classification of hyponatremia type was assigned using a multi-stage algorithm, which has not yet been validated. Accordingly, it is possible that patients excluded from the analysis due to hypovolemic hyponatremia may have been erroneously excluded. It should be further noted that assignment of disease progression was based on modified RECIST 1.1 and Cheson criteria and study results may vary from clinical − trial-based protocols.

## Conclusion

It has been shown that the incidence of hyponatremia is high, not only in lung cancer, but also in patients with lymphoma, BC, and CRC. Additionally, the occurrence of hyponatremia in all four types of cancer is associated with poorer OS. An awareness of hyponatremia in cancer is important as it is commonly underestimated by oncologists due to the difficulty of its interpretation [4]. Further studies are warranted to explore the effects of correction of hyponatremia on survival in cancer patients.

## Abbreviations

BC, breast cancer; CRC, colorectal cancer; ECOG, Eastern Cooperative Oncology Group; HR, hazard ratio; IDN, integrated delivery network; IR, incidence rate; IRB, institutional review board; KPS, Karnofsky Performance Status; NSCLC, non-small cell lung cancer; OS, overall survival; PFS, progression-free survival; PS, performance status; SCLC, small cell lung cancer; SD, standard deviation; SIADH, syndrome of inappropriate antidiuretic hormone secretion
